# Identification and Antifungal Activity of Compounds from the Mangrove Endophytic Fungus *Aspergillus clavatus* R7

**DOI:** 10.3390/md15080259

**Published:** 2017-08-19

**Authors:** Wensheng Li, Ping Xiong, Wenxu Zheng, Xinwei Zhu, Zhigang She, Weijia Ding, Chunyuan Li

**Affiliations:** 1 College of Materials and Energy, South China Agricultural University, Guangzhou 510642, China; wenshengscau@126.com (W.L.); xp0000542003@scau.edu.cn (P.X.); wzheng@scau.edu.cn (W.Z.); m15521182580@163.com (X.Z.); 2 School of Chemistry and Chemical Engineering, Sun Yat-Sen University, Guangzhou 510275, China; cesshzhg@mail.sysu.edu.cn

**Keywords:** mangrove endophytic fungus, coumarin, chromone, sterone, antifungal activity, *Aspergillusclavatus*

## Abstract

Two new coumarin derivatives, 4,4′-dimethoxy-5,5′-dimethyl-7,7′-oxydicoumarin (**1**), 7-(γ,γ-dimethylallyloxy)-5-methoxy-4-methylcoumarin (**2**), a new chromone derivative, (*S*)-5-hydroxy-2,6-dimethyl-4*H*-furo[3,4-g]benzopyran-4,8(6*H*)-dione (**5**), and a new sterone derivative, 24-hydroxylergosta-4,6,8(14),22-tetraen-3-one (**6**), along with two known bicoumarins, kotanin (**3**) and orlandin (**4**), were isolated from an endophytic fungus *Aspergillus* *clavatus* (collection No. R7), isolated from the root of *Myoporum bontioides* collected from Leizhou Peninsula, China. Their structures were elucidated using 1D- and 2D- NMR spectroscopy, and HRESIMS. The absolute configuration of compound **5** was determined by comparison of the experimental and calculated electronic circular dichroism (ECD) spectra. Compound **6** significantly inhibited the plant pathogenic fungi *Fusarium oxysporum*, *Colletotrichum musae* and *Penicillium italicum*, compound **5** significantly inhibited *Colletotrichum musae*, and compounds **1**, **3** and **4** greatly inhibited *Fusarium oxysporum*, showing the antifungal activities higher than those of the positive control, triadimefon.

## 1. Introduction

**Figure 1:**
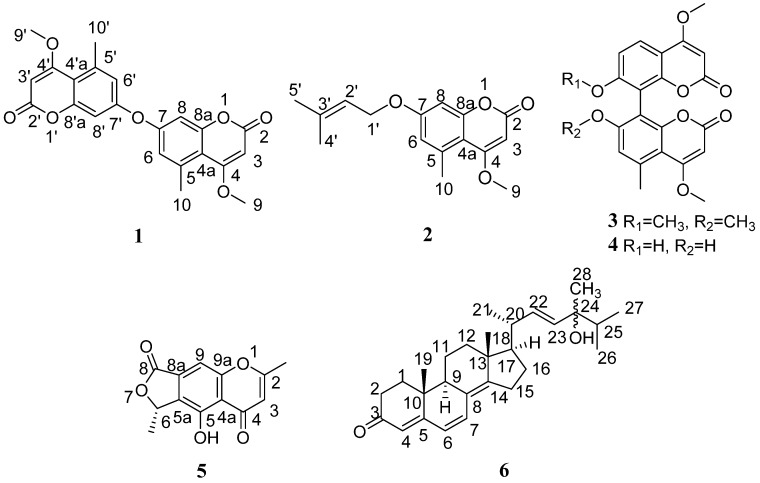
The chemical structures of compounds **1**–**6**.

Marine mangrove endophytic fungi are among the most productive sources of structurally unusual and biologically active natural products [1,2,3]. *Aspergillus clavatus*, belonging to Ascomycetes (Eurotiales, Trichocomaceae), is usually found as a saprophytic fungus, which is widespread in nature, producing mycotoxins and other metabolites with activities [4,5,6,7,8,9]. In our continuous search for new bioactive natural products from mangrove endophytes, the methanol extract from the endophytic fungus, *A. clavatus* (collection No. R7) isolated from the root of *Myoporum bontioides* A. Gray collected from Leizhou Peninsula, China, had been screened to show antifungal activities against several plant pathogenic fungi [10]. This prompted us to investigate the corresponding metabolites. As a result, two new coumarin derivatives, 4,4′-dimethoxy-5,5′-dimethyl-7,7′-oxydicoumarin (**1**), 7-(γ,γ-dimethylallyloxy)-5-methoxy-4-methylcoumarin (**2**), a new chromone derivative, (*S*)-5-hydroxy-2,6-dimethyl-4*H*-furo[3,4-g]benzopyran-4,8(6*H*)-dione (**5**), and a new sterone derivative, 24-hydroxylergosta-4,6,8(14),22-tetraen-3-one (**6**), along with two known bicoumarins, kotanin (**3**) and orlandin (**4**) [11], were isolated (Figure 1). Herein, we report their isolation, structural elucidation and bioactivity.

## 2. Results and Discussion

**Figure 2:**
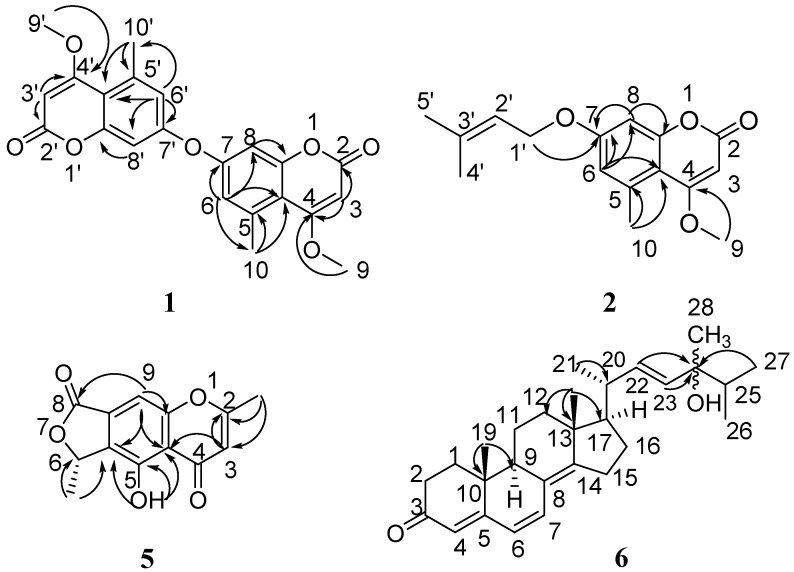
Selected HMBC (arrow) correlations of **1**, **2**, **5** and **6**.

Compound **1** was obtained as a white, amorphous powder. It showed a molecular ion peak at *m/z* 395.1129 in the positive HR-ESI-MS spectrum, corresponding to molecular formula C_22_H_18_O_7_ (fourteen degrees of unsaturation) ([M + H]^+^, calcd. 395.1125). The ^1^H NMR spectrum of **1** (Table 1) exhibited signals of two meta-coupling aromatic protons at δ_H_ 6.92 (d, 1H, 2.4 Hz) and 7.05 (d, 1H, 2.4 Hz), an olefinic proton at δ_H_ 5.70 (s, 1H), an aromatic methyl group at δ_H_ 2.56 (s, 3H) and a methoxyl group at δ_H_ 3.94 (s, 3H). The ^13^C NMR and HSQC spectra of **1** revealed 11 carbon signals, including one methyl, one methoxyl, one ester carbonyl and eight olefinic carbons. These NMR and MS data suggested that compound **1** was most likely a symmetrical coumarin dimer derivative [12,13], wherein each subunit was substituted by one methoxyl and one methyl, and connected together by one oxygen atom. Comparison of the NMR spectral data of compound **1** with those of the known 7-hydroxy-4-methoxy-5-methylcoumarin [11] showed great similarity in that they both use deuterated dimethyl sulfoxide as solvent. However, the chemical shifts of compound **1** are obviously shifted downfield by 3.3/0.28, 6.0/0.37 ppm at C-6/H-6, C-8/H-8, and upfield by 5.7 ppm at C-7, compared with those of 7-hydroxy-4-methoxy-5-methylcoumarin, suggesting that the two coumarin subunits were presumably connected together through an oxygen atom from C-7 and C-7′ in **1**. This presumption was further confirmed by HMBC experiment (Figure 2). HMBC correlations from δ_H_ 6.92 (H-6/H-6′) to δ_C_ 109.4 (C-4a/C-4′a), 155.5 (C-7/C-7′), 105.4 (C-8/C-8′) and 23.5 (C-10/C-10′), from δ_H_ 2.56 (H-10/H-10′) to δ_C_ 109.4 (C-4a/C-4′a), 137.9 (C-5/C-5′) and 119.6 (C-6/C-6′), and from δ_H_ 7.05 (H-8/H-8′) to δ_C_ 109.4 (C-4a/C-4′a) and 156.4 (C-8a/C-8′a), suggested that the methyl (C-10/C-10′) and the oxygen atom were attached on C-5/C-5′ and C-7/C-7′, respectively. Simultaneously, HMBC correlations from δ_H_ 5.70 (H-3/H-3′) to δ_C_ 162.1 (C-2/C-2′), 169.5 (C-4/C-4′), and 109.4 (C-4a/C-4′a), and from δ_H_ 3.94 (H-9/H-9′) to 169.5 (C-4/C-4′), along with a four-bond HMBC correlation from δ_H_ 2.56 (H-10/H-10′) to δ_C_ 169.5 (C-4/C-4′), indicated that the methoxyl was connected to C-4/C-4′. Therefore, compound **1** was unambiguously elucidated as 4,4′-dimethoxy-5,5′-dimethyl-7,7′-oxydicoumarin.

Compound **2** was obtained as white needles. Its molecular formula of C_16_H_18_O_4_ (eight degrees of unsaturation) was determined based on HRESIMS (*m/z* 275.1277 [M + H]^+^, calcd. 275.1277, and 297.1106 [M + Na]^+^, calcd. 297.1097). The ^1^H NMR spectrum (Table 1) showed signals of two meta-coupling aromatic protons at δ_H_ 6.64 (d, 1H, 2.4 Hz) and 6.68 (d, 1H, 2.4 Hz), an olefinic proton at δ_H_ 5.54 (s, 1H), an aromatic methyl group at δ_H_ 2.62 (s, 3H), a methoxyl group at δ_H_ 3.94 (s, 3H), and a prenyloxy moiety at δ_H_ 1.77 (3H, s), 1.82 (3H, s), 5.47 (1H, t, 7.2 Hz), 4.55 (d, 2H, 7.2 Hz). The ^13^C NMR spectrum (Table 1) exhibited 16 carbon including one methyl, one methoxyl, one ester carbonyl, one prenyl group, and eight olefinic carbons. These NMR data of **2** were similar to those of 7-hydroxy-4-methoxy-5-methylcoumarin [11]. The obvious difference between them was ascribed to a prenyl group of the former replaced the hydroxyl proton of the latter. This deduction and the position of the prenyloxy group in **2** was confirmed by comparision with the reported examples of 7-*O*-prenyl coumarins such as marianins A, B [14], and anisocoumarin B [15], and by HMBC (Figure 2) correlations from H-1′ to C-7, from H-6 to C-7, C-8, C-4a, C-10, and from H-8 to C-6, C-7, C-4a and C-8a. Additionally, the positions of the other two substituents were confirmed to be the same as 7-hydroxy-4-methoxy-5-methylcoumarin by detailed analysis of the HMBC spectrum. Thus, the structure of 4 was elucidated as 7-(γ,γ-dimethylallyloxy)-5-methoxy-4-methylcoumarin.

**Figure 3:**
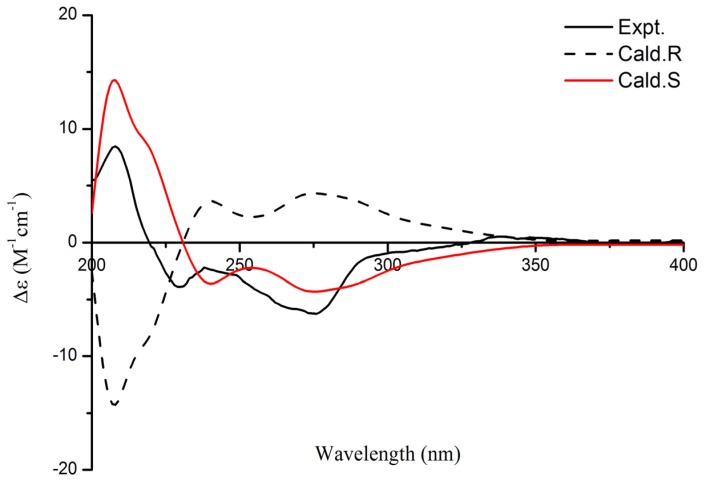
The calculated and experimental ECD spectra of **5**.

Compound **5** was obtained as colorless powders, and its molecular formula was established as C_13_H_10_O_5_ with nine degrees of unsaturation by positive HR-ESI-MS (*m/z* 269.0423, [M + Na]^+^, calcd. 269.0420). The characteristic UV absorption maxima at 229, 242, 263, 345 nm suggested the presence of a chromone pattern in **5** [16,17]. The ^1^H and ^13^C NMR spectral data of **5** are listed in Table 2. The ^1^H NMR spectrum exhibited signals of one olefinic methyl at δ_H_ 2.52 (s, 1H), one secondary methyl at δ_H_ 1.67 (d, 6.6 Hz 3H) connected to one oxomethine at δ_H_ 5.73 (q, 6.6 Hz, 1H), one hydroxyl at δ_H_ 13.43 (s, 1H), and two aromatic proton singlets at δ_H_ 6.37 and 7.83. The olefinic methyl was revealed to be attached at C-2 due to HMBC correlations (Figure 2) from the 2-CH_3_ proton at δ_H_ 2.52 to C-2 (δ_C_ 170.3) and C-3 (δ_C_ 108.9), and from the aromatic H-3 proton (δ_H_ 6.37) to C-2 and C-4a (δ_C_ 112.7). The hydroxyl was proved to be substituted at C-5 based on HMBC correlations from 5-OH (δ_H_ 13.43) to C-4a, C-5 (δ_C_ 155.8) and C-5a (δ_C_ 130.7). These results, combined with the HMBC correlations, including H-9 (δ_H_ 7.37) to C-4a, C-5a, and the oxygen-bearing C-9a (δ_C_ 157.2), ambiguously established the chromone substructure, indicating that the positions of C-8a and C-9 of the aromatic ring were substituted by the remaining moiety. Subsequently, HMBC correlations from H-9 to C-8 (δ_C_ 168.2), from H-6 (δ_H_ 5.73) to C-5a, C-8, 6-CH_3_ (δ_H_ 1.67), from 6-CH_3_ to C-5a, C-6 (δ_C_ 76.5), together with the remaining 2 degrees of unsaturation revealed by the molecular formula, suggested a γ-valerolactone ring system attached to C-8a and C-9 through C-8 and C-6, respectively. Thus, the planar structure of **5** was completely established. The absolute configuration of **5** was determined by comparing the theoretical calculation of ECD (electronic circular dichroism) with the experimental ECD [18,19]. The experimental ECD of **5** is similar to the ECD of the (*S*)-model compound (Figure 3), so as to determine that the absolute configuration of **5** was 6*S*. Therefore, the structure of **5** was as shown in Figure 1.

Compound **6** was obtained as colorless needles. The molecular formula was determined as C_28_H_40_O_2_ (nine degrees of unsaturation) by analysis of positive HR-ESI-MS (*m/z* 409.3108; [M + H]^+^, calcd. 409.3101). The ^1^H NMR spectrum of **6** displayed five olefinic proton signals at δ_H_ 6.04 (d, 1H, 9.6 Hz), 6.61 (d, 1H, 9.6 Hz), 5.75 (s, 1H), 5.48 (m, 1H), 5.49 (m, 1H), six methyl signals at δ_H_ 0.98 (s, 3H), 1.00 (s, 3H), 1.08 (d, 3H, 6.6 Hz), 0.91 (d, 3H, 3.1 Hz), 0.90 (d, 3H, 3.2 Hz), 1.23 (s, 3H), and numerous methene and methine signals ranging from *δ*_H_ 1.29 to 2.53. The ^13^C NMR and HSQC spectra showed 28 carbons, including a ketone group (δ_C_ 199.5), and four olefinic double bonds. Comparison of the ^1^H and ^13^C NMR spectral data (Table 3) of compound **6** with those of ergosta-4,6,8(14),22-tetraen-3-one [20], revealed their great structural similarities. However, the ^13^C NMR of the former exhibited one quaternary carbon more at δ_C_ 74.9 and one methine less at high field than in the latter. This result combined the difference between their molecular formulas presumed **6** to be a hydroxyl substituted derivate of ergosta-4,6,8(14),22-tetraen-3-one. The position of the hydroxyl group was confirmed to locate at C-24 by HMBC correlations of H-22, H-23, H-26, H-27 and H-28 to C-24 at δ_C_ 74.9. Detailed analysis of HSQC, ^1^H-^1^H-COSY, and HMBC spectra (Figure 2) allowed the complete assignment of the proton and carbon signals of **6**. The relative configuration of **6** was assigned by NOESY (nuclear overhauser effect spectroscopy) experiments. In the NOESY spectrum of **6**, NOE (nuclear overhauser effect) correlations of Me-18 with both H-20 and H-11a, and the lack of NOE correlations between Me-18 and H-11b, suggested β-orientations of Me-18, H-20 and H-11a. Consequently, NOE correlations between H-19 and H-11a, and the absence of NOE correlations between H-19 and H-11b suggested H-19 was also in a β-orientation. Additionally, NOE correlations between H-9 and H-1a, and between H-19 and H-1b, along with no NOE correlations between H-19 and H-1a, indicated H-9 was in an *α*-orientation. The configuration of double bond Δ22 was deduced to be *E* by comparison of the chemical shifts with those of the same positions of ergosta-4,6,8(14),22-tetraen-3-one and the large coupling constant (15.2 Hz) between H-22 and H-23. The configuration of C-24 could not be assigned based on the obtained NOE data. Therefore, compound **6** was elucidated as 24-hydroxylergosta-4,6,8(14),22-tetraen-3-one, as shown in Figure 1.

In addition, the structures of the known compounds **3** and **4** [11] were identified by comparison of their spectroscopic data with those reported in the literature. HRESIMS, ^1^H, ^13^C, ^1^H-^1^H COSY, HSQC and HMBC NMR spectra of the new compounds are available at the Supplementary Materials File (Figures S1–S22).

The antifungal activities of the isolated compounds were examined in vitro towards three plant pathogens, including *Fusarium oxysporum* Schlecht. f. sp. lycopersici (Sacc.) W.C. Snyder et H.N. Hansen (*F. oxysporum*), *Colletotrichum musae* (Berk. and M. A. Curtis) Arx. (*C. musae*), and *Penicillium italicum* Wehme (*P. italicm*). From the results presented in Table 4, all of the compounds showed broad-spectrum inhibitory activities against these fungi except compound **2**, which is inactive towards *P. italicm* with MIC value >729.66 μM. Moreover, compound **6** exhibited the strongest broad-spectrum inhibitory activities against all the three pathogenic fungi *F. oxysporum*, *C. musae* and *P. italicm* with MIC values of 244.73, 195.79 and 61.18 μM, respectively, in comparison with other compounds and triadimefon (used as the positive control, MIC values = 340.43, 272.39, 170.24 μM, respectively). In addition, compounds **1**, **3** and **4** showed high activities against *F. oxysporum* (MICs = 253.81, 235.85, 252.47 μM, respectively), which was better than triadimefon. Whereas compound **5** displayed more potent inhibitory activity against *C. musae*, with MIC values of 203.07 μM, than triadimefon.

## 3. Experimental Section

### 3.1. General Experimental Procetures

Melting points were determined using a JH30 melting point detector (Jia Hang Instrument Co., Ltd., Shanghai, China). Optical rotations were measured using a Horiba SEPA-300 polarimeter at 25 °C. The UV spectra were obtained on a Shimadzu UV-2550 spectrophotometer (Shimadzu, Tokyo, Japan), and IR spectra were run on a Nicolet 5DX-Fourier transform infrared spectrophotometer (Thermo Electron Corporation, Madison, WI, USA). NMR spectra data were recorded at Bruker AV-600 MHz NMR spectrometers (Bruker Biospin AG, Fällanden, Switzerland), with tetramethylsilane (TMS) as internal standard, and the chemical shifts were reported in δ values (ppm). The HRESIMS spectra were recorded on an Q-TOF mass spectrometer (Thermo Fisher, Frankfurt, Germany). CD spectra were recorded with a Chirascan™ CD spectrometer (Applied Photophysics, Leatherhead, UK). Silica gel (200–300 mesh) for column chromatography was purchased from Qingdao Haiyang Chemical Co., Ltd., Qingdao, China. Sephadex LH-20 was purchased from Amersham Pharmacia Biotech. Buckinghamshire, UK. All other chemicals were of analytical grade.

### 3.2. Fungal Material and Fermentation

The fungal strain R7 was isolated from the root of *M. bontioides*, collected from the mangrove in Leizhou peninsula, China, in May 2014, and deposited at the College of Materials and Energy, South China Agricultural University, Guangdong Province, China. The strain has been identified as *A. clavatus*, according to morphologic traits and molecular identification [10]. Its 599 base pair ITS sequence had 99% sequence identity to those of several *A. clavatus* strains (AY373847.1, NR121482.1, KF669481.1) by a NCBI BLAST search. The sequence data has been submitted to GenBank with accession number KY765893.

A small agar scrap with mycelium of the fungal isolate which was grown on potato dextrose agar medium for 5 days at 28 °C was added into 250 mL GYT medium (1% glucose, 0.1% yeast extract, 0.2% peptone, 0.2% crude sea salt), and incubated at 28 °C, 180 rpm for 6 days as seed culture. Then the seed culture was grown on a solid autoclaved rice substrate medium (one hundred 1000 mL Erlenmeyer flasks, each containing 100 mL water, 100 g rice and 0.3 g crude sea salt) for 30 days at 25 °C under static stations.

### 3.3. Extraction and Isolation

The mycelia and solid rice medium were extracted with 95% ethanol three times. The solvent was concentrated to 1 L *in vacuo* and extracted with equal volume of ethyl acetate, yielding 70.0 g extract. Then the extract was subjected to a silica gel column (30 × 6 cm), eluting with gradient of petroleum ether/ethyl acetate (97:3, 95:5, 75:25, 50:50, 25:75, 0:100, *v*/*v*) to afford six fractions (Fr. A1–Fr. A6). Fraction A2 was chromatographed on Sephadex LH-20 CC (110 × 4 cm) eluting with Methanol-dichloromethane-petroleum ether (2:2:1, *v/v*), to obtain three subfractions (Fr. A2-1–Fr. A2-3) based on TLC properties. Fraction A2-3 was dissolved in acetone and recrystallized at room temperature to afford compound **5** (8.2 mg). Fraction A3 was purified by preparative silica gel TLC (petroleum ether/ethyl acetate, 5:1, *v/v*) to yield compound **6** (15 mg). Fraction A4 was further fractioned by silica gel eluting with petroleum ether-ethyl acetate (85:15, 75:25, 50:50, *v/v*) to give three subfractions (Fr. A4-1–Fr. A4-3). Fraction A4-1 was separated through Sephadex LH-20 CC (methanol-dichloromethane 3:2, *v/v*) to afford compound **2** (7.5 mg). Fraction A4-3 was applied to preparative silica gel TLC (petroleum ether/ethyl acetate, 1:5, *v/v*) to give compounds **3** (6.8 mg) and **4** (4.3 mg). Fraction A6 was subjected to silica gel column chromatography and eluted with ethyl acetate/methanol (50:50, 15:85, 0:100, *v/v*), leading to three subfractions (Fr. A6-1–Fr. A6-3). Fraction A6-3 was further chromatographed on a Sephadex LH-20 column using methanol/dichloromethane (3:2, *v/v*) to afford compound **1** (1.8 mg).

*4,4′-dimethoxy-5,5′-dimethyl-7,7′-oxydicoumarin* (**1**): White amorphous powder. m.p. 174.7–175.3 °C; HR-ESI-MS *m/z* 315.1129 ([M + H]^+^, calcd. for C_22_H_1__9_O_7_ 315.1125). ^1^H NMR and ^13^C NMR data see Table 1.

*7-(γ,γ-dimethylallyloxy)-5-methoxy-4-methylcoumarin* (**2**): White crystal. m.p. 115.5–116.3 °C*;* UV (EtOH) λ_max_ (log ε): 208 (4.28), 218 (4.08), 310 (3.81) nm; IR (KBr) ν_max_: 3144, 2968, 1716, 1613, 1575, 1400, 1256, 1152 cm^−1^; HR-ESI-MS *m/z* 275.1127 ([M + H]^+^, calcd. for C_16_H_1__9_O_4_ 275.1127). ^1^H NMR and ^13^C NMR see Table 1.

*(S)-5-hydroxy-2,6-dimethyl-4H-furo[3,4-g]benzopyran-4,8(6H)-dione* (**5**): White needles. [α]_D_^25^ = −37.87 (c 0.0015, MeOH); UV (MeOH) λ_max_ (log ε): 229 (4.32), 242 (4.24), 263 (3.72), 345 (3.63) nm; IR (KBr) ν_max_: 3420, 2987, 1635, 1616, 1487, 1396, 1173 cm^−1^; HR-ESI-MS *m/z* 269.0423 ([M + Na]^+^, calcd. for C_13_H_10_O_5_Na 269.0420). ^1^H NMR and ^13^C NMR see Table 2.

*24-hydroxylergosta-4,6,8(14),22-tetraen-3-one* (**6**): Yellow oil. [α]_D_^25^ = +173.3 (c 0.004, MeOH); UV (MeOH) λ_max_ (log ε): 341 (3.79) nm; IR (KBr) ν_max_: 3420, 3136, 1669, 1650, 1528, 1453, 1401, 1385 cm^−1^; HR-ESI-MS *m/z* 409.3108 ([M + H]^+^, calcd. for C_28_H_4__1_O_2_ 409.3101). ^1^H NMR and ^13^C NMR see Table 3.

### 3.4. Computational Analyses

Conformational analyses for compound **5** were performed via Spartan’10 software (Wavefunction, Inc., Irvine, CA, USA) using the MMFF94 molecular mechanics force field calculation. Conformers within a 10 kcal/mol energy window were generated and optimized using DFT calculations at the B3LYP/6-31G (d) level. Conformers for R or S were chosen for ECD calculations in MeOH at the B3LYP/6-311+G (2d, p) level. Rotary strengths for a total of 50 excited states were calculated. The IEF-PCM solvent model for MeOH was used. The calculated ECD spectra were obtained by density functional theory (DFT) and time-dependent DFT (TD-DFT) using Gaussian 09 (Gaussian Inc., Wallingford, CT, USA) program package. The calculated ECD curve was generated using SpecDis 1.6 software package (University of Wurzburg, Wurzburg, Germany) with a half-bandwidth of 0.2 eV.

### 3.5. Antifungal Activity Assay

The following four phytopathogenic fungi were used for bioassay: *F. oxysporum*, *C. musae*, and *P. italicm*. They were obtained from the College of Agriculture, South China Agricultural University. The antifungal activities of the isolated compounds were determined by the broth dilution method as described in the previous report to get the minimum inhibitory concentration (MIC) [21]. Triadimefon and the solvent were used as positive and negative control, respectively.

## 4. Conclusions

In conclusion, two new coumarin derivatives, 4,4′-dimethoxy-5,5′-dimethyl-7,7′-oxydicoumarin (**1**), 7-(γ,γ-dimethylallyloxy)-5-methoxy-4-methylcoumarin (**2**), a new chromone derivative, (*S*)-5-hydroxy-2,6-dimethyl-4*H*-furo[3,4-g]benzopyran-4,8(6*H*)-dione(**5**), and a new sterone derivative, 24-hydroxylergosta-4,6,8(14),22-tetraen-3-one (**6**), together with two known bicoumarins, kotanin (**3**) and orlandin (**4**), were isolated from an endophytic fungus *Aspergillus clavatus* R7, isolated from the root of *Myoporum bontioides* that collected from Leizhou Peninsula, China. Compound **6** remarkably inhibited *Fusarium oxysporum*, *Colletotrichum musae* and *Penicillium italicum*, compound **5** highly inhibited *Colletotrichum musae*, and compounds **1**, **3** and **4** greatly inhibited *Fusarium oxysporum*, by comparison to triadimefon, indicating that these compounds could be used as leads of new fungicides.

## Figures and Tables

**Table 1:** ^1^H and ^13^C NMR data for compounds **1** and **2**.

No.	1 ^a^		2 ^b^	
	δ_C_	δ_H_, Mult. (*J* in Hz)	δ_C_	δ_H_, Mult. (*J* in Hz)
1				
2	162.1, C	7.61, s	163.2, C	
2-OH				
3	88.6, CH	5.7, s	87.5, CH	5.54, s
4	169.5, C		169.8, C	
5	137.9, C		138.4, C	
6	119.6, CH	6.92, d (2.4)	116.3, CH	6.64, d (2.4)
7	155.5, C		161.2, C	
8	105.4, CH	7.05, d (2.4)	99.4, CH	6.68, d (2.4)
9	57.2, CH_3_	3.94, s	55.9, CH_3_	3.94, s
10	23.5, CH_3_	2.56, s	23.4, CH_3_	2.62, s
4a	109.4, C		107.8, C	
8a	156.4, C		156.6, C	
1′			65.1, CH_2_	4.55, d (7.2)
2′	162.1, C	7.61, s	118.8, CH	5.47, t (7.2)
3′	88.6, CH	5.7, s	139.1, C	
4′	169.5, C		25.8, CH_3_	1.82, s
5′	137.9, C		18.2, CH_3_	1.77, s
6′	119.6, CH	6.92, d (2.4)		
7′	155.5, C			
8′	105.4, CH	7.05, d (2.4)		
9′	57.2, CH_3_	3.94, s		
10′	23.5, CH_3_	2.56, s		
4′a	109.4, C			
8′a	156.4, C			

^a^ Measured in CD_3_COCD_3_; ^b^ Measured in CDCl_3_.

**Table 2:** ^1^H and ^13^C NMR data for compound **5** in CD_3_COCD_3_.

No.	δ_C_	δ_H_, Mult. (*J* in Hz)
1		
2	170.3, C	
2-CH_3_	19.7, CH_3_	2.52, s
3	108.9, CH	6.37, s
4	184.0, C	
4a	112.7, C	
5	155.8, C	
5-OH		13.43, s
5a	130.7, C	
6	76.5, CH	5.73, q (6.6)
6-CH_3_	18.2, CH_3_	1.67, d (6.6)
8	168.2, C	
8a	131.0, C	
9	102.9, CH	7.37, s
9a	157.2, C	

**Table 3:** ^1^H and ^13^C NMR data for compound **6** in CDCl_3_.

No.	δ_C_	δ_H_, Mult. (*J* in Hz)
1	34.1, CH_2_	a1.82, m b2.02, m
2	34.1, CH_2_	a2.46, m b2.53, m
3	199.5, C	
4	123.0, CH	5.75, s
5	124.5, C	
6	124.6, CH	6.04, d (9.6)
7	134.0, CH	6.61, d (9.6)
8	164.3, C	
9	44.3, CH	2.14, m
10	36.7, C	
11	19.0, CH_2_	a1.60, m b1.71, m
12	35.6, CH_2_	a1.31, m b2.09, m
13	44.0, C	
14	155.7, C	
15	25.2, CH_2_	a2.39, m b2.48, m
16	27.8, CH_2_	a1.49, m b1.80, m
7	55.9, CH	1.29, m
18	19.0, CH_3_	0.98, s
19	16.6, CH_3_	1.00, s
20	39.1, CH	2.22, m
21	21.0, CH_3_	1.08, d (6.6)
22	133.7, CH)	5.48, dd (8.2, 15.2)
23	134.0, CH	5.52, d (15.2)
24	74.9, C	
25	38.1, CH	1.70, m
26	17.6, CH_3_	0.91, d (3.1)
27	17.2, CH_3_	0.90, d (3.2)
28	25.4, CH_3_	1.23, s

**Table 4:** Antifungal activity of the isolated compounds by MIC values (µM).

Compounds	*F. oxysporum*	*C. musae*	*P. italicm*
**1**	253.81	380.71	253.81
**2**	729.66	547.25	>729.66
**3**	235.85	353.77	235.85
**4**	252.47	378.71	252.47
**5**	609.21	203.07	304.61
**6**	244.73	195.79	61.18
Triadimefon ^a^	340.43	272.39	170.24

^a^ positive control.

## Supplementary Materials

The NMR and MS spectra of **1**, **2**, **5** and **6** are available online at www.mdpi.com/link/1660-3397/15/8/259/s1 in Figures S1–S22.
