# Supplementary material for: Identification and Antifungal Activity of Compounds from the Mangrove Endophytic Fungus Aspergillus clavatus R7
Source: Mar Drugs. 2017 Aug 19;15(8):259. doi: 10.3390/md15080259 (PMC5577613; doi:10.3390/md15080259)
Supplement: Supplementary file 1 [file marinedrugs-15-00259-s001.pdf]

Supplementary Materials: Identification and antifungal activity of compounds from the mangrove endophytic fungus *Aspergillus clavatus* R7

Wensheng Li [1], Ping Xiong [1], Wenxu Zheng [1], Xinwei Zhu [1], Zhigang She [2], Weijia Ding [1,*] and Chunyuan Li [1,*]

[1] College of Materials and Energy, South China Agricultural University, Guangzhou 510642, China; wenshengscau@126.com (W.L.); xp0000542003@scau.edu.cn (P.X.); wzheng@scau.edu.cn (W.Z.); m15521182580@163.com (X.Z.); dwjzsu@scau.edu.cn (W.D); chunyuan-li@163.com (C.L.)

[2] School of Chemistry and Chemical Engineering, Sun Yat-sen University, Guangzhou 510275, China; cesshzhg@mail.sysu.edu.cn (Z.S.)

* Correspondence: dwjzsu@scau.edu.cn (W.D.); chunyuan-li@163.com (C.L.); Tel.: +86-20-85280319 (C.L.)

Contents

Figure S1. ^{1}H NMR spectrum (600 MHz) of compound **1** in DMSO S2

Figure S2. ^{13}C NMR spectrum (150 MHz) of compound **1** in DMSO S2

Figure S3. HSQC spectrum (600/150 MHz) of compound **1** in DMSO S3

Figure S4. HMBC spectrum (600/150 MHz) of compound **1** in DMSO S3

Figure S5. HRESIMS spectrum of compound **1**. S4

Figure S6. ^{1}H NMR spectrum (600 MHz) of compound **2** in $CDCl_3$ S4

Figure S7. ^{13}C NMR spectrum (600 MHz) of compound **2** in $CDCl_3$ S5

Figure S8. HSQC spectrum (600/150 MHz) of compound **2** in $CDCl_3$ S5

Figure S9. HMBC spectrum (600/150 MHz) of compound **2** in $CDCl_3$ S6

Figure S10. HRESIMS spectrum of compound **2** S6

Figure S11. ^{1}H NMR spectrum (600 MHz) of compound **5** in CD_3COCD_3 .... S7

Figure S12. ^{13}C NMR spectrum (150 MHz) of compound **5** in CD_3COCD_3 .... S7

Figure S13. HSQC spectrum (600/150 MHz) of compound **5** in CD_3COCD_3 .... S8

Figure S14. HMBC spectrum (600/150 MHz) of compound **5** in CD_3COCD_3 .... S8

Figure S15. HRESIMS spectrum of compound **5** S9

Figure S16. ^{1}H NMR spectrum (600 MHz) of compound **6** in $CDCl_3$ S9

Figure S17. ^{13}C NMR spectrum (600 MHz) of compound **6** in $CDCl_3$. S10

Figure S18. HSQC spectrum (600/150 MHz) of compound **6** in $CDCl_3$ S10

Figure S19. HMBC spectrum (600/150 MHz) of compound **6** in $CDCl_3$ S11

Figure S20. ^{1}H-^{1}HCOSY spectrum (600 MHz) of compound 6 in $CDCl_3$ S11

Figure S21. NOESY spectrum (600 MHz) of compound **6** in $CDCl_3$. S12

Figure S22. HRESIMS spectrum of compound **6** S12

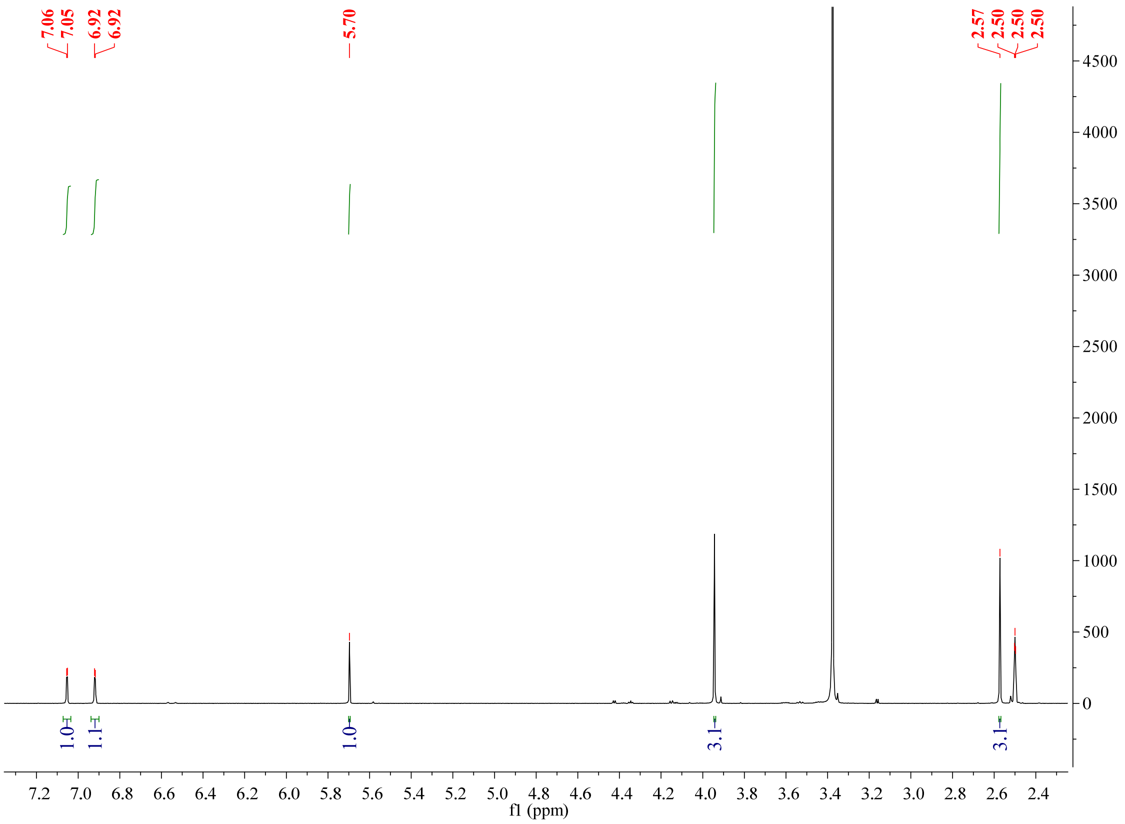

Figure S1. ^{1}H NMR spectrum (600 MHz) of compound **1** in DMSO.

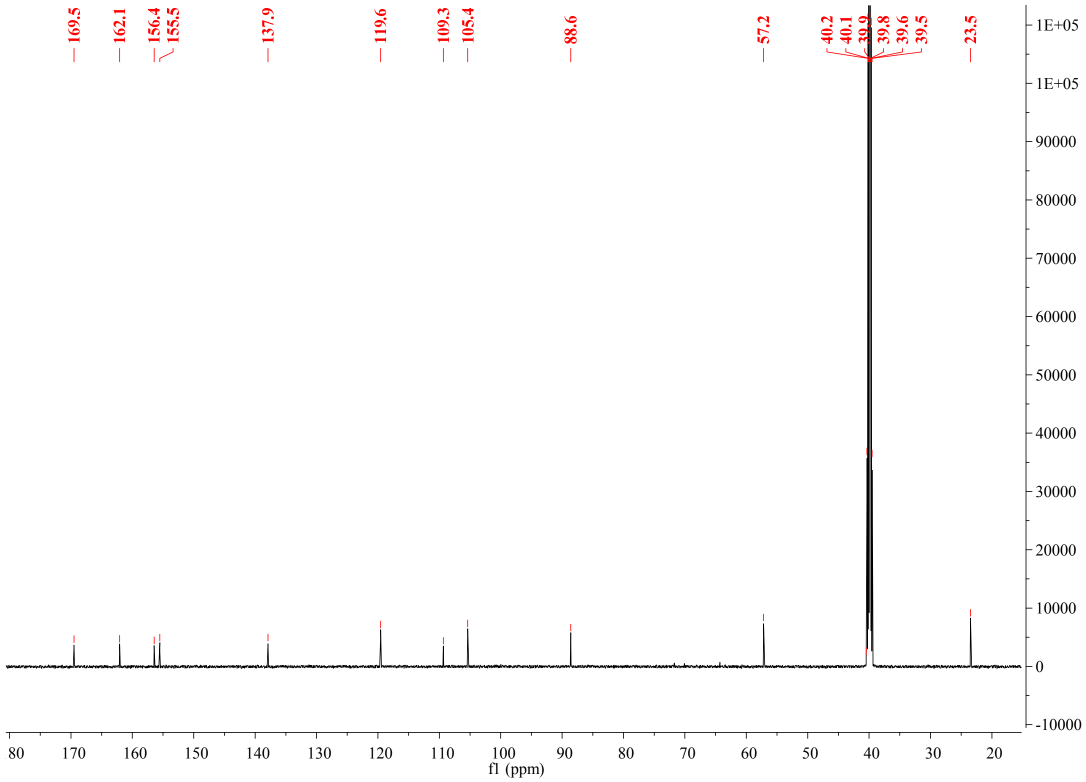

Figure S2. ^{13}C NMR spectrum (150 MHz) of compound **1** in DMSO

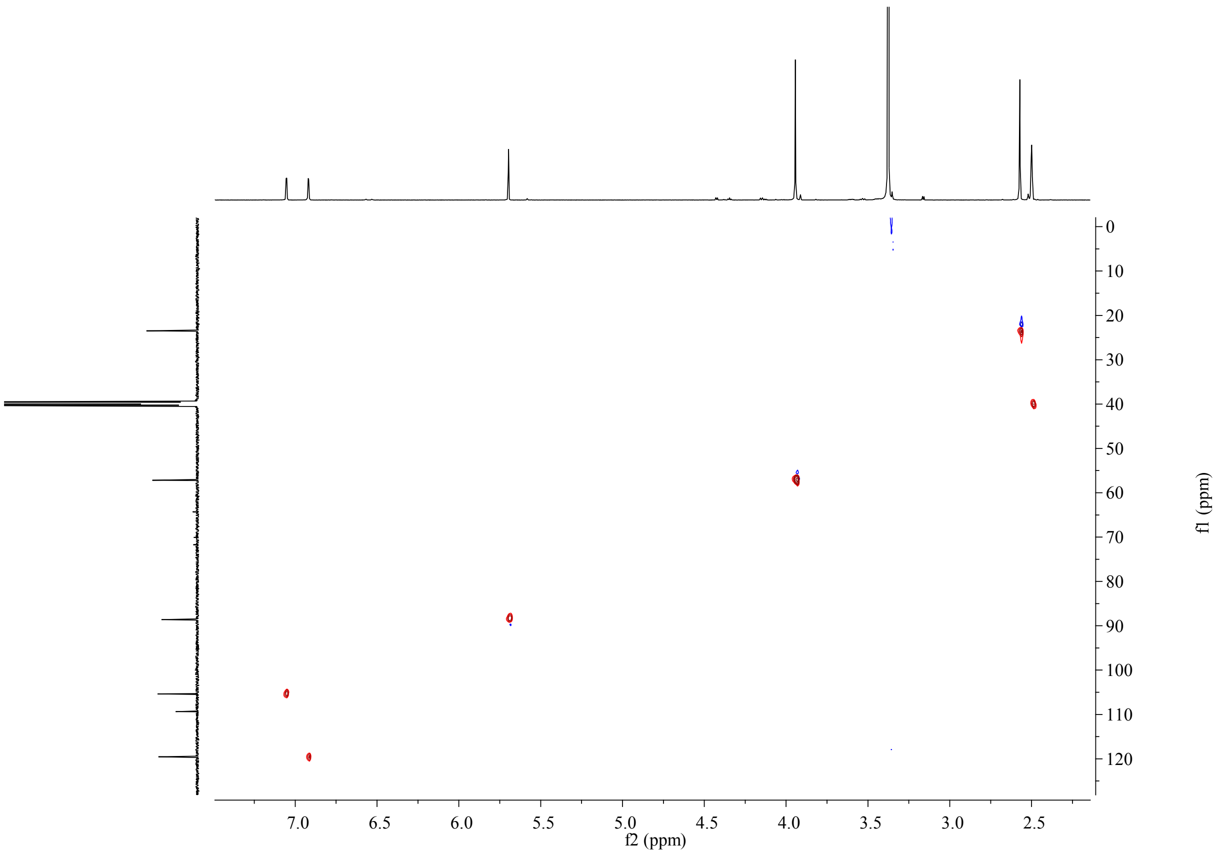

Figure S3. HSQC spectrum (600/150 MHz) of compound **1** in DMSO

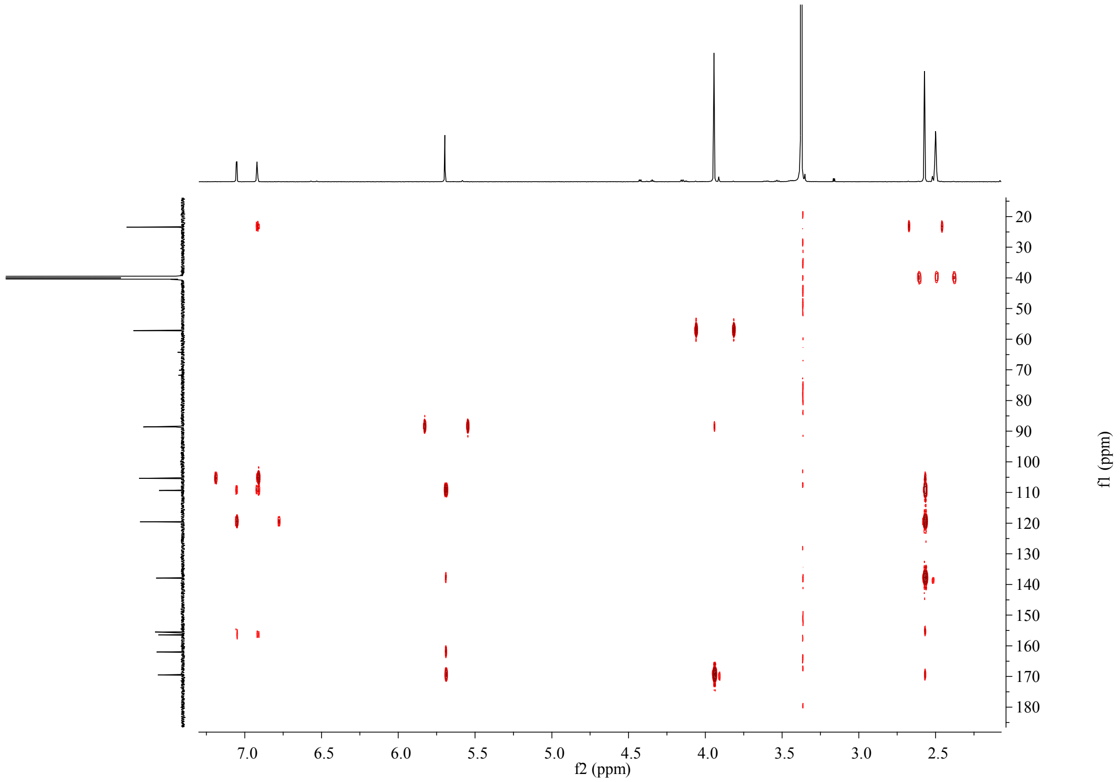

Figure S4. HMBC spectrum (600/150 MHz) of compound **1** in DMSO.

Generic Display Report

Analysis Info

Analysis Name	D:\Data\amaZon\MS\data\2016\12\lws_kczn11_pos.d	Acquisition Date	12/28/2016 3:36:56 PM
Method	Default for Dicrect Infusion.m	Operator	SCSIO
Sample Name	lijun_AM_EtOAc	Instrument	amaZon SL
Comment			

Acquisition Parameter

Source Type	ESI	Ion Polarity	Positive	Set Nebulizer	0.4 Bar
Focus	Active	Set Capillary	4500 V	Set Dry Heater	180 °C
Scan Begin	100 m/z	Set End Plate Offset	-500 V	Set Dry Gas	4.0 l/min
Scan End	2000 m/z	Set Collision Cell RF	800.0 Vpp	Set Divert Valve	Waste

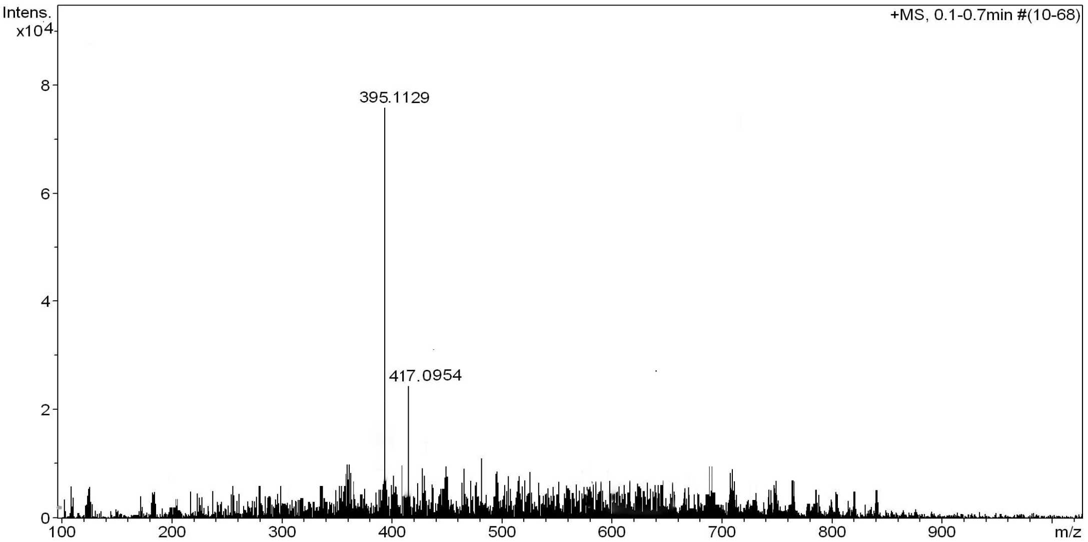

Meas. m/z	#	Formula	Score	m/z	err [ppm]	err [mDa]	mSigma	rdb	e⁻ Conf	N-Rule
395.1129	1	C 22 H 19 O 7	100.00	395.1125	0.8	0.4	26.9	13.5	even	ok
417.0954	1	C 22 H 18 Na O 7	100.00	417. 0950	1.3	0.6	15.3	13.5	even	ok

Figure S5. HRESIMS spectrum of compound **1**.

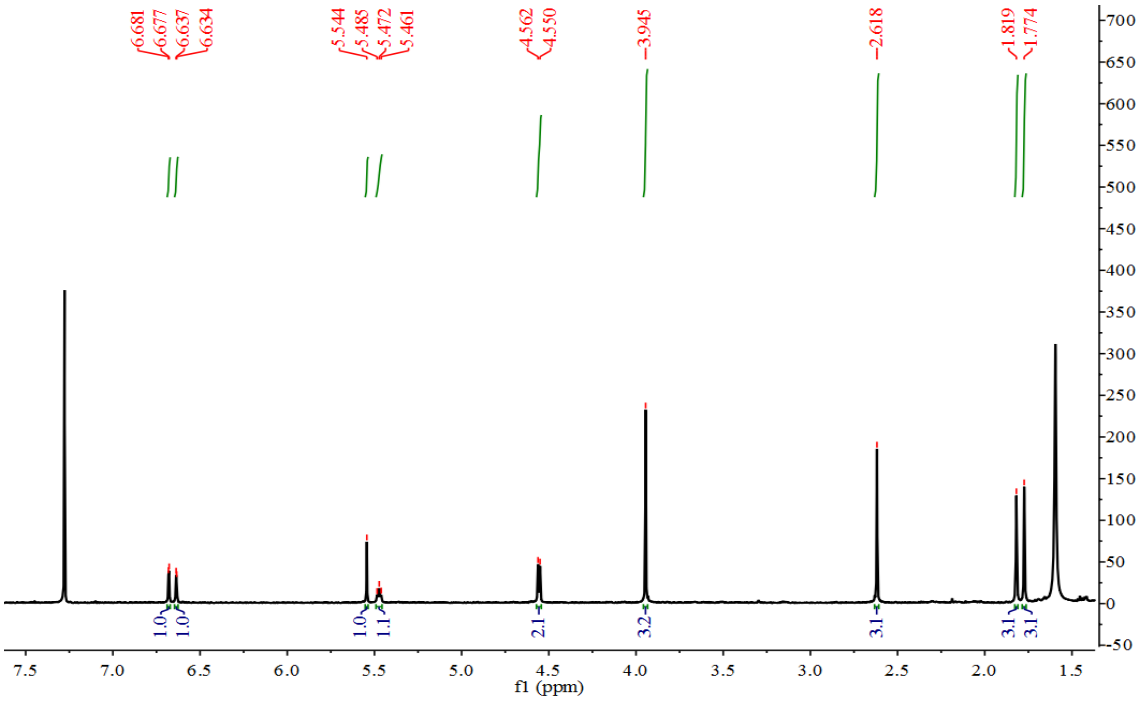

Figure S6. ^{1}H NMR spectrum (600 MHz) of compound **2** in $CDCl_3$.

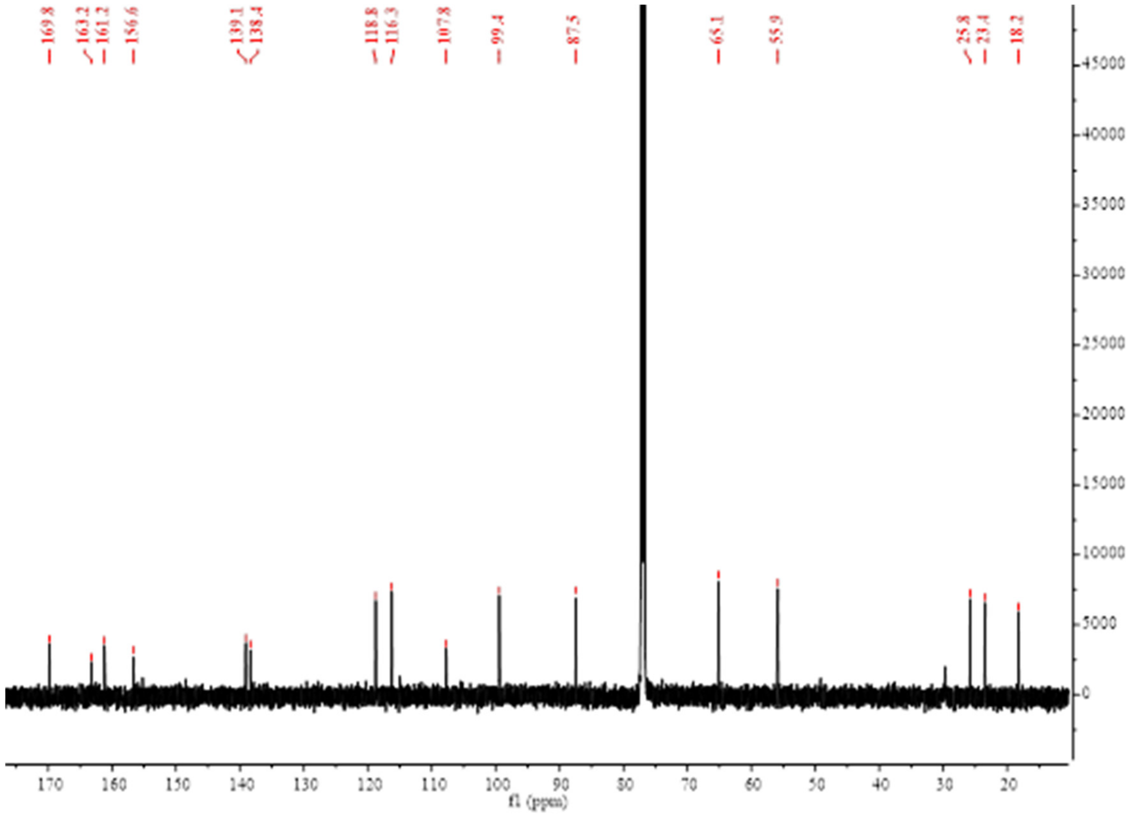

Figure S7. ^{13}C NMR spectrum (600 MHz) of compound **2** in $CDCl_3$.

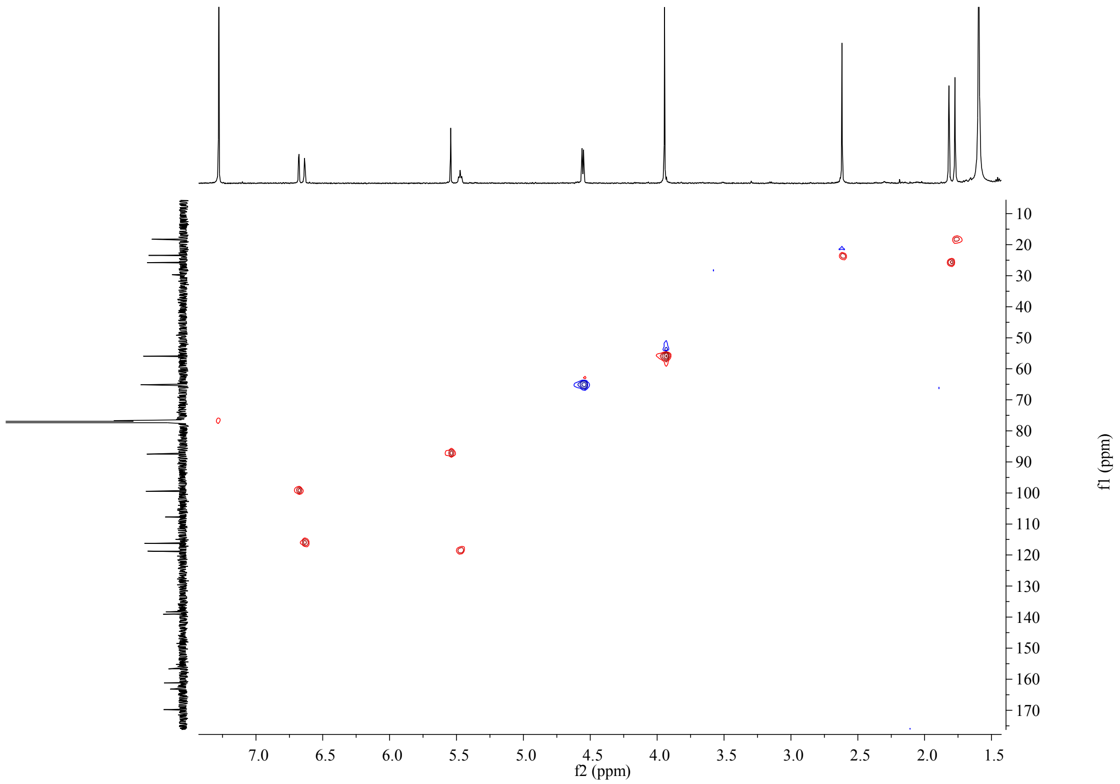

Figure S8. HSQC spectrum (600/150 MHz) of compound 2 in $CDCl_3$.

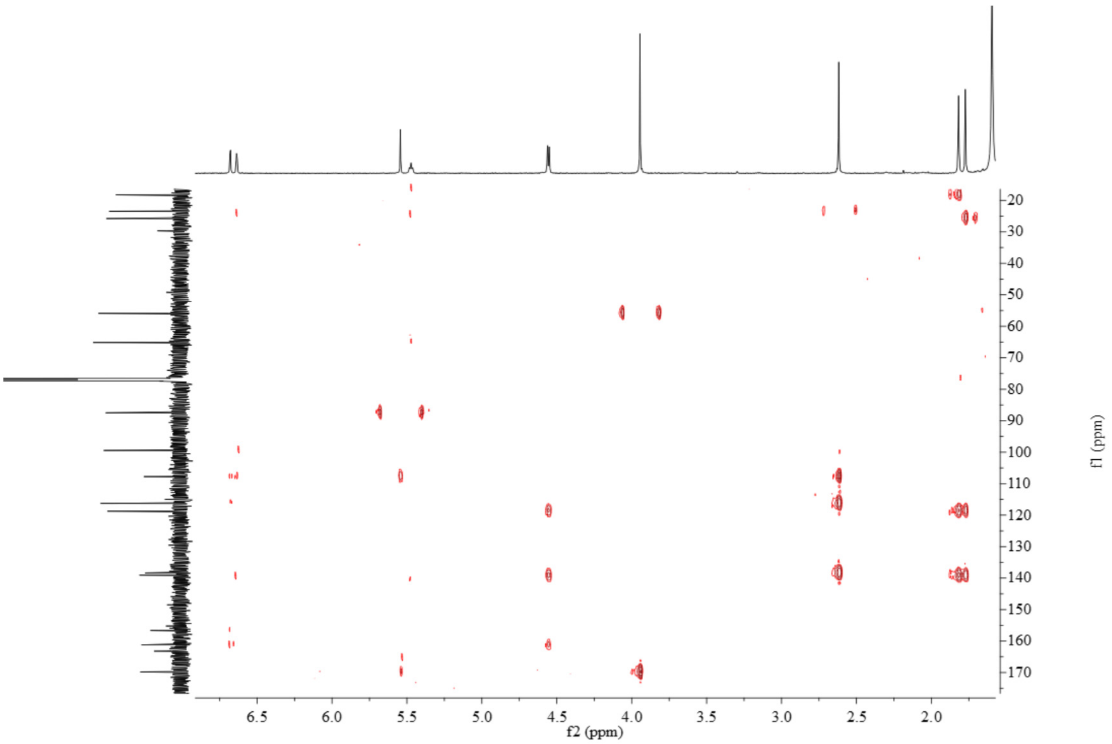

Figure S9. HMBC spectrum (600/150 MHz) of compound 2 in $CDCl_3$.

Mass Spectrum SmartFormula Report

Analysis Info

		Acquisition Date	6/29/2016 9:51:33 AM
Analysis Name	D:\Data\MS\data\201606\liwensheng_kcn-18f_pos_1_01_2072.d		
Method	LC_Direct Infusion_pos_100-1000mz.m	Operator	SCSIO
Sample Name	liwensheng_kcn-18f_pos	Instrument	maXis 255552.00029
Comment			

Acquisition Parameter

Source Type	ESI	Ion Polarity	Positive	Set Nebulizer	0.4 Bar
Focus	Active	Set Capillary	4500 V	Set Dry Heater	180 °C
Scan Begin	100 m/z	Set End Plate Offset	-500 V	Set Dry Gas	4.0 l/min
Scan End	2000 m/z	n/a	n/a	Set Divert Valve	Waste
		Set Corona	0 nA	Set APCI Heater	0 °C

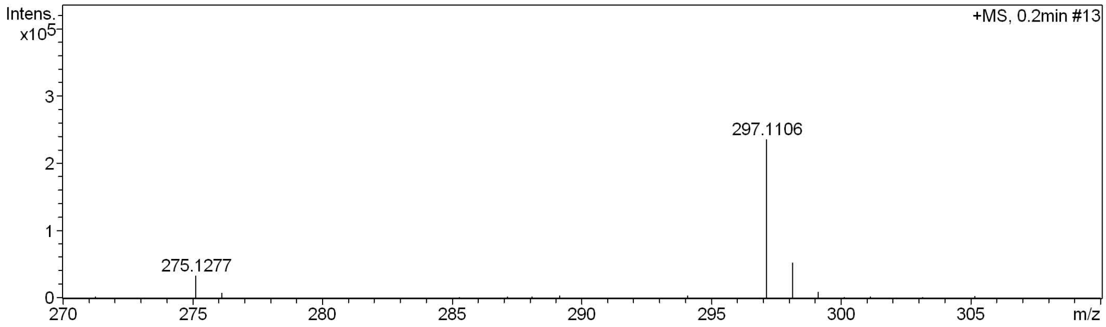

Meas. m/z	#	Ion Formula	Score	m/z	err [ppm]	err [mDa]	mSigma	rdb	e⁻ Conf	N-Rule
275.127743	1	C16H19O4	100.00	275.127786	0.2	0.0	34.2	7.5	even	ok
297.110629	1	C16H18NaO4	100.00	297.109730	3.0	0.9	27.9	7.5	even	ok
571.232300	1	C32H36NaO8	26.77	571.230239	-3.6	-2.1	30.7	14.5	even	ok

Figure S10. HRESIMS spectrum of compound **2**.

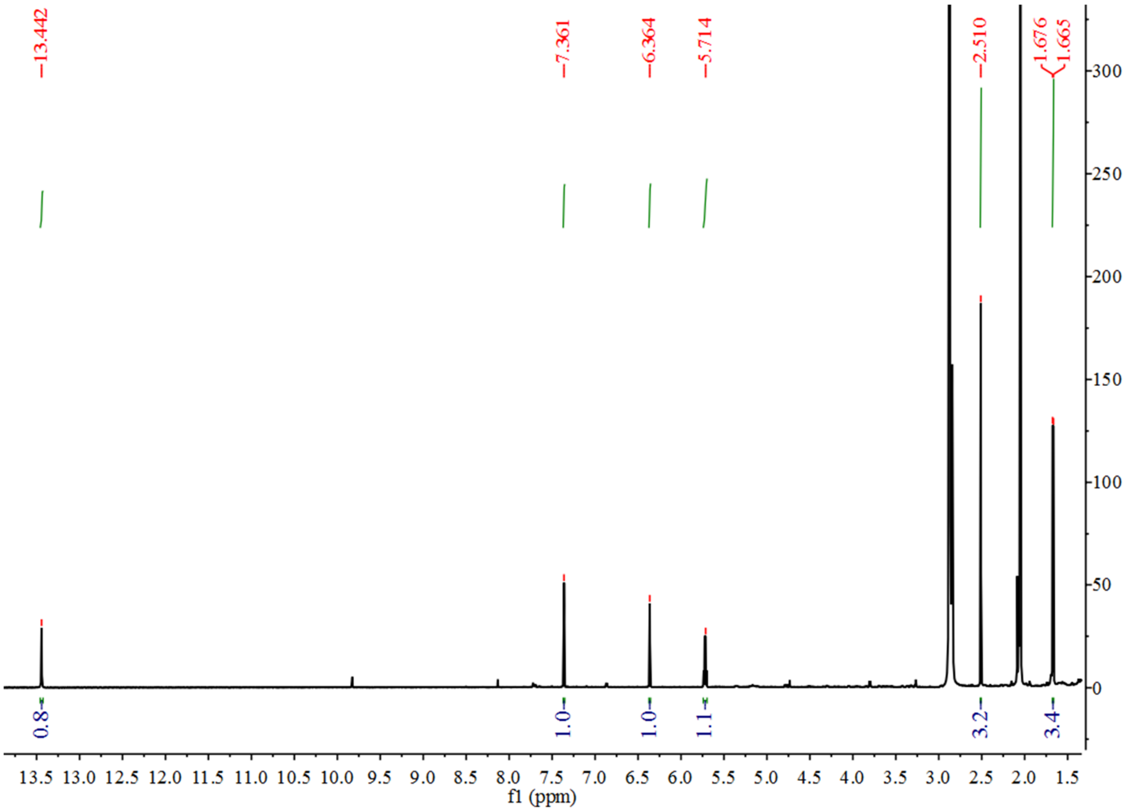

Figure S11. ^{1}H NMR spectrum (600 MHz) of compound **5** in CD_3COCD_3.

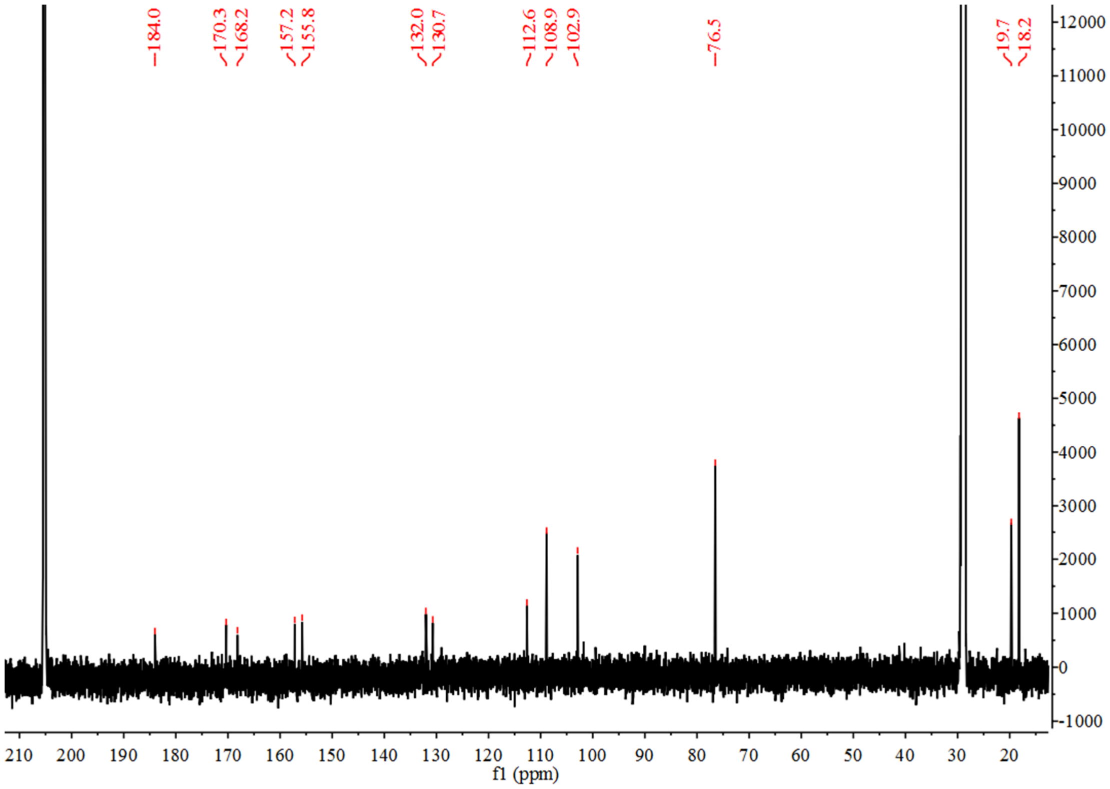

Figure S12. ^{13}C NMR spectrum (150 MHz) of compound **5** in CD_3COCD_3.

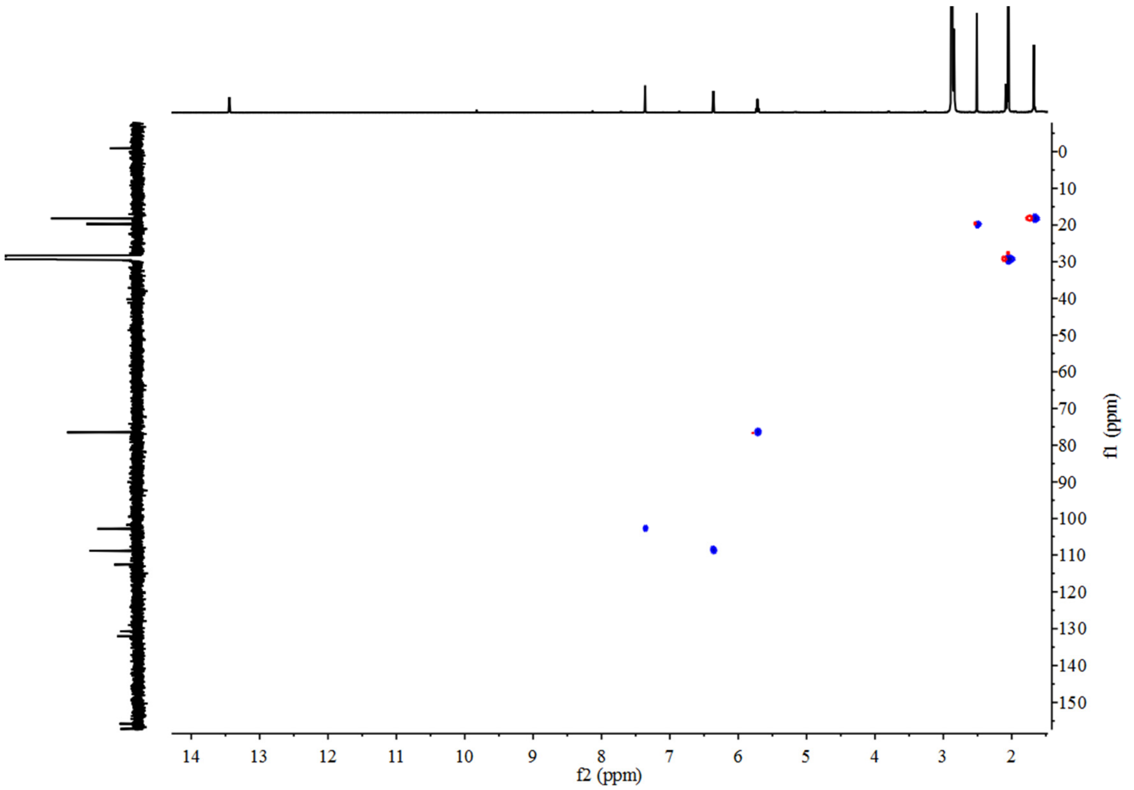

Figure S13. HSQC spectrum (600/150 MHz) of compound **5** in CD3COCD3.

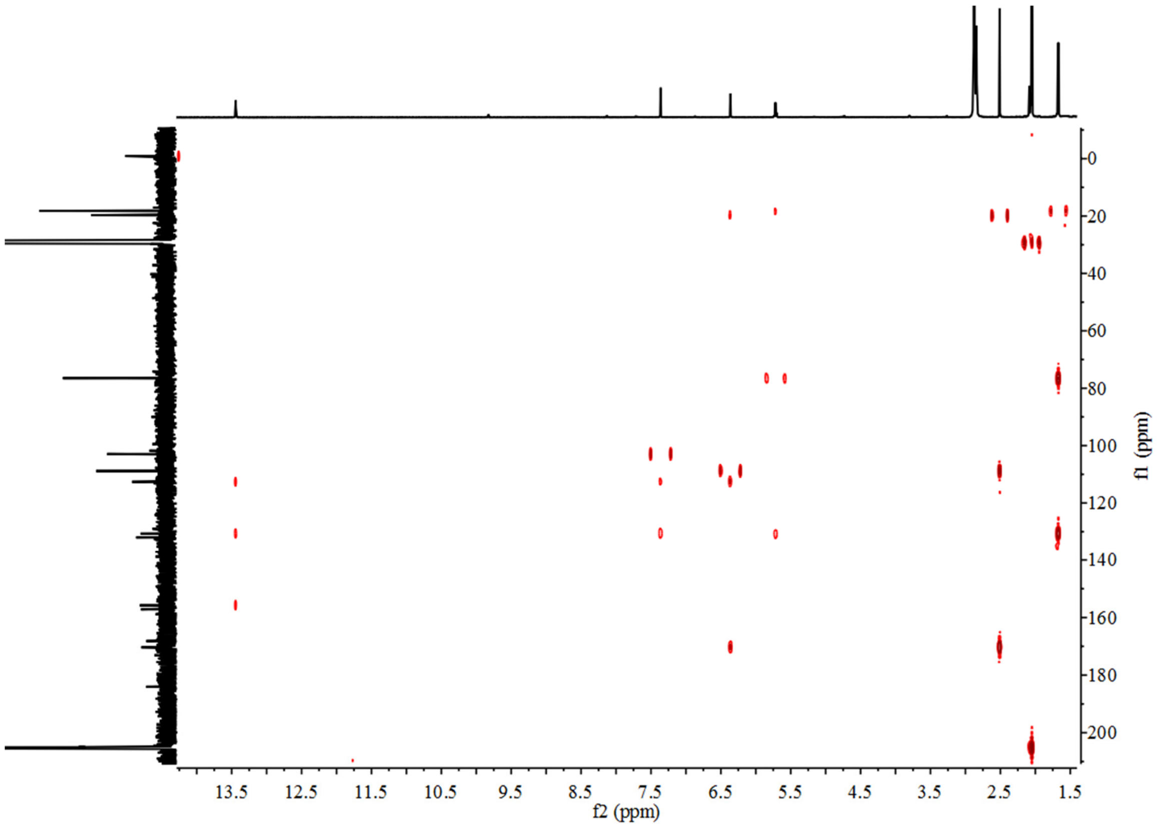

Figure S14. HMBC spectrum (600/150 MHz) of compound **5** in CD3COCD3.

Mass Spectrum SmartFormula Report

Analysis Info

Analysis Name	D:\Data\MS\data\201611\zhuxinwei_ZH-3_pos_34_01_2603.d	Acquisition Date	11/15/2016 4:24:54 PM
Method	LC_Direct Infusion_pos_100-1000mz.m	Operator	SCSIO
Sample Name	zhuxinwei_ZH-3_pos	Instrument / Ser#	maXis 29
Comment			

Acquisition Parameter

Source Type	ESI	Ion Polarity	Positive	Set Nebulizer	0.4 Bar
Focus	Active	Set Capillary	4500 V	Set Dry Heater	180 °C
Scan Begin	100 m/z	Set End Plate Offset	-500 V	Set Dry Gas	4.0 l/min
Scan End	2000 m/z	Set Collision Cell RF	800.0 Vpp	Set Divert Valve	Waste

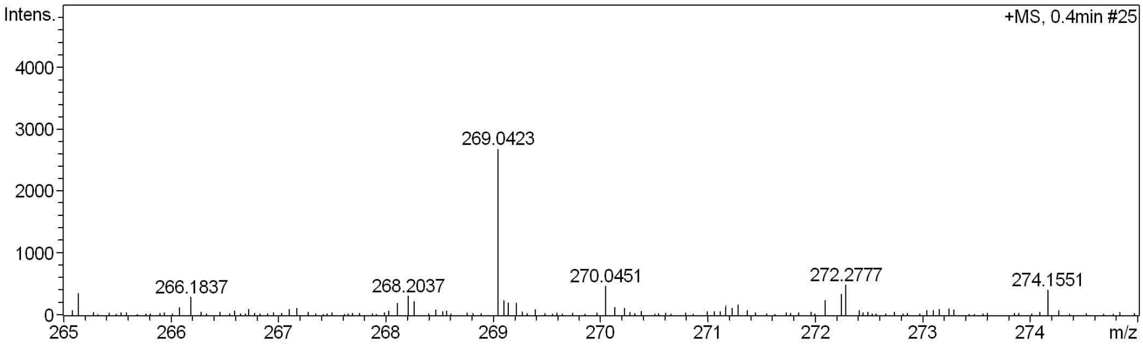

Meas. m/z	#	Formula	Score	m/z	err [ppm]	err [mDa]	mSigma	rdb	e⁻ Conf	N-Rule
269.0423	1	C 13 H 10 Na O 5	100.00	269.0420	-1.0	-0.3	22.8	8.5	even	ok

Figure S15. HRESIMS spectrum of compound **5**.

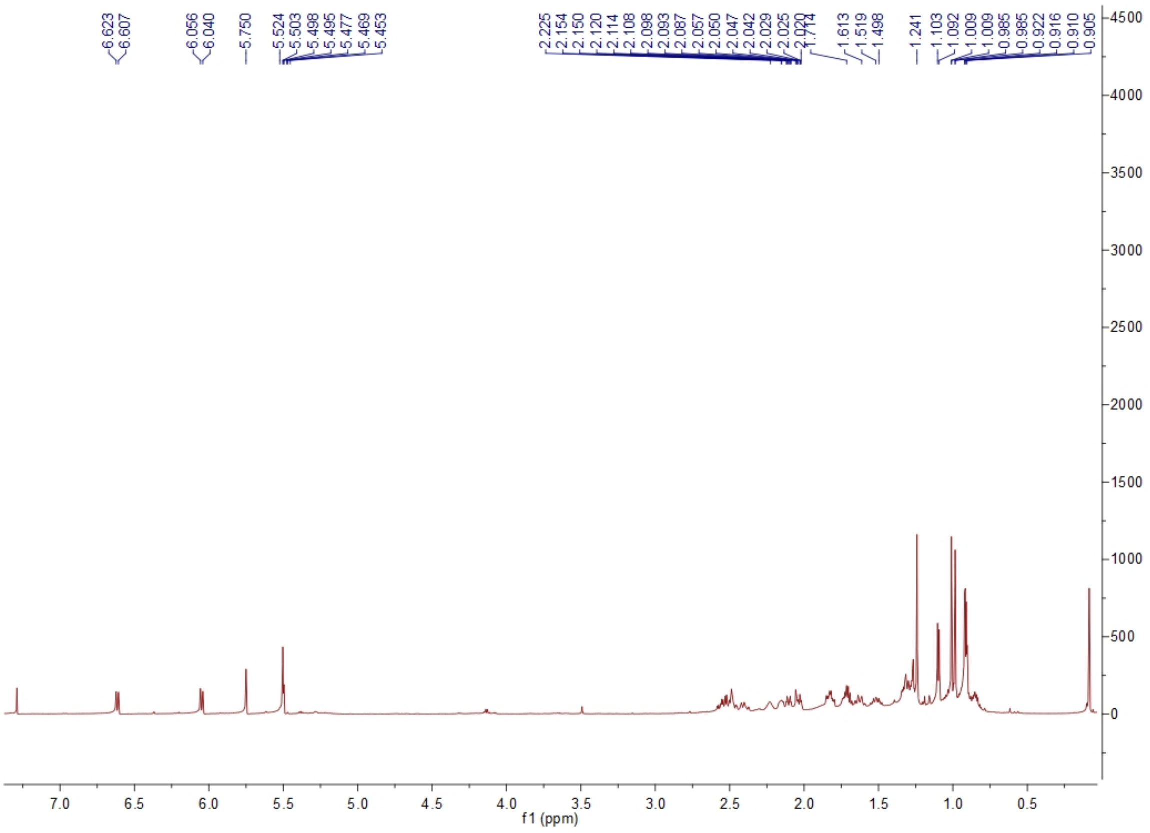

Figure S16. ^{1}H NMR spectrum (600 MHz) of compound **6** in $CDCl_3$.

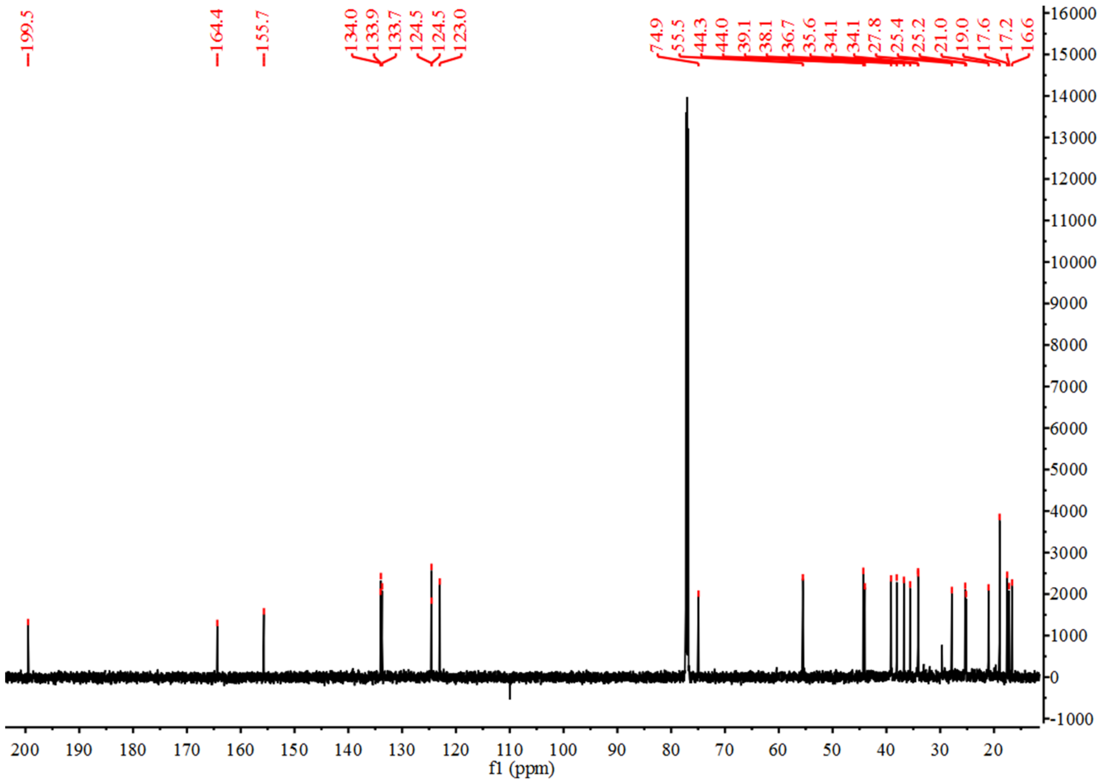

Figure S17. ^{13}C NMR spectrum (600 MHz) of compound **6** in $CDCl_3$.

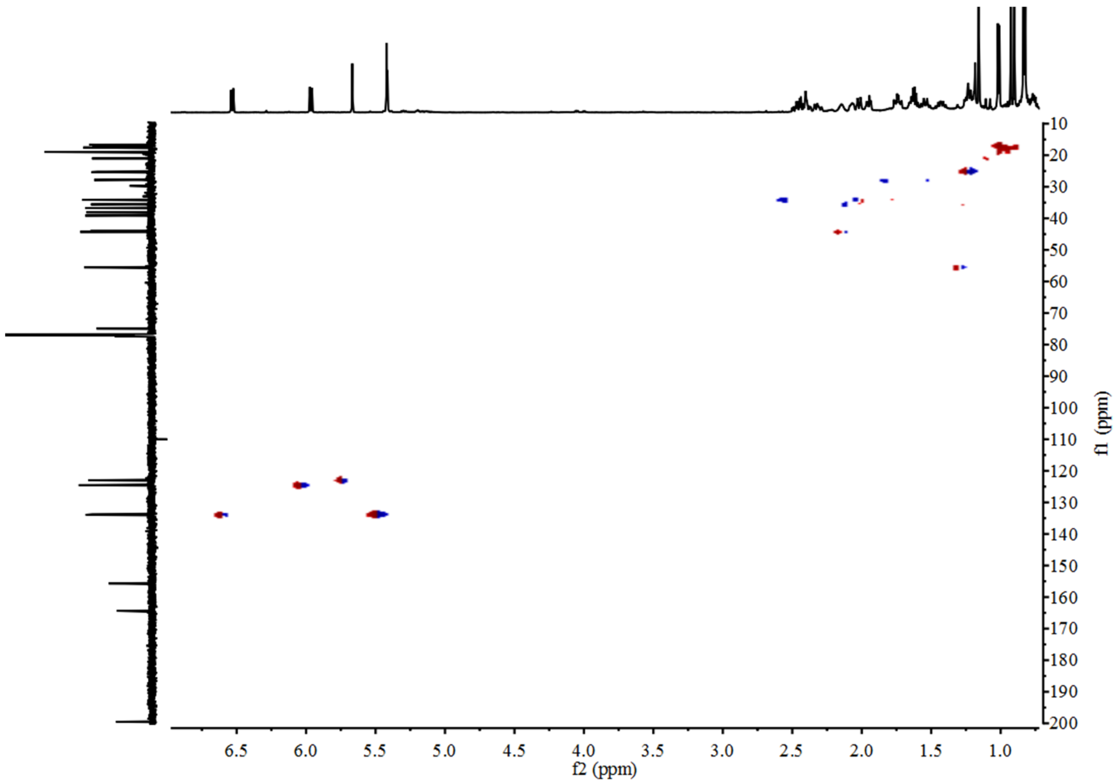

Figure S18. HSQC spectrum (600/150 MHz) of compound **6** in $CDCl_3$

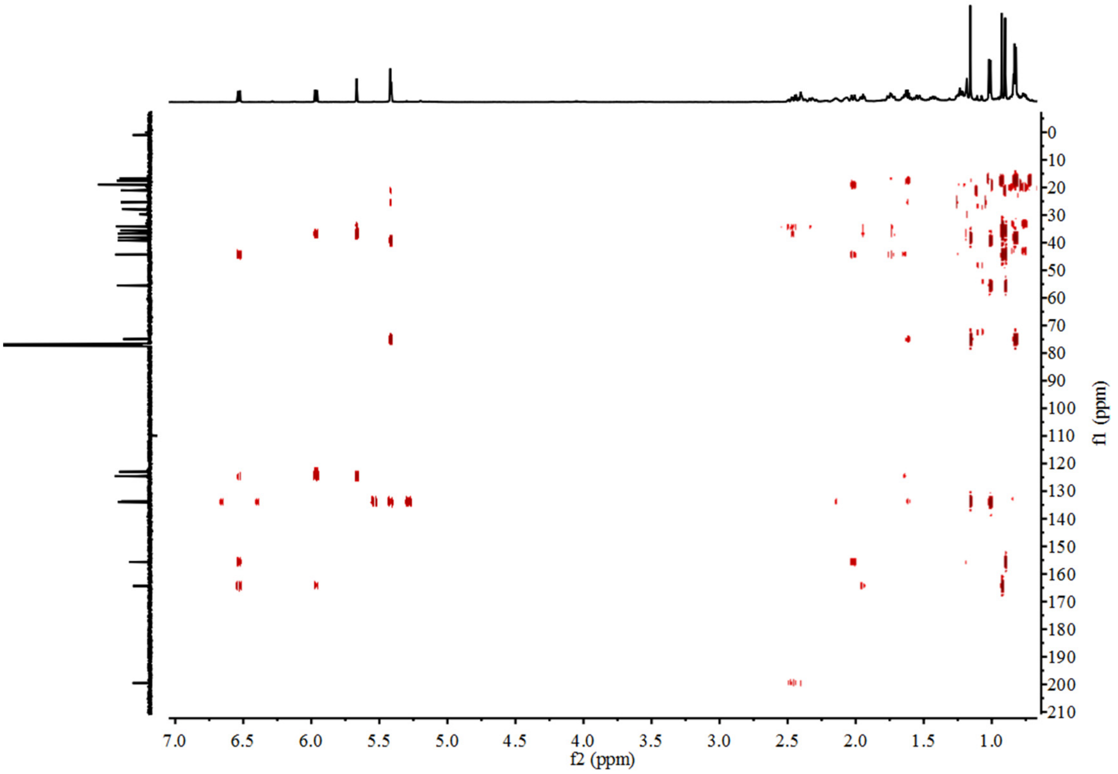

Figure S19. HMBC spectrum (600/150 MHz) of compound **6** in $CDCl_3$.

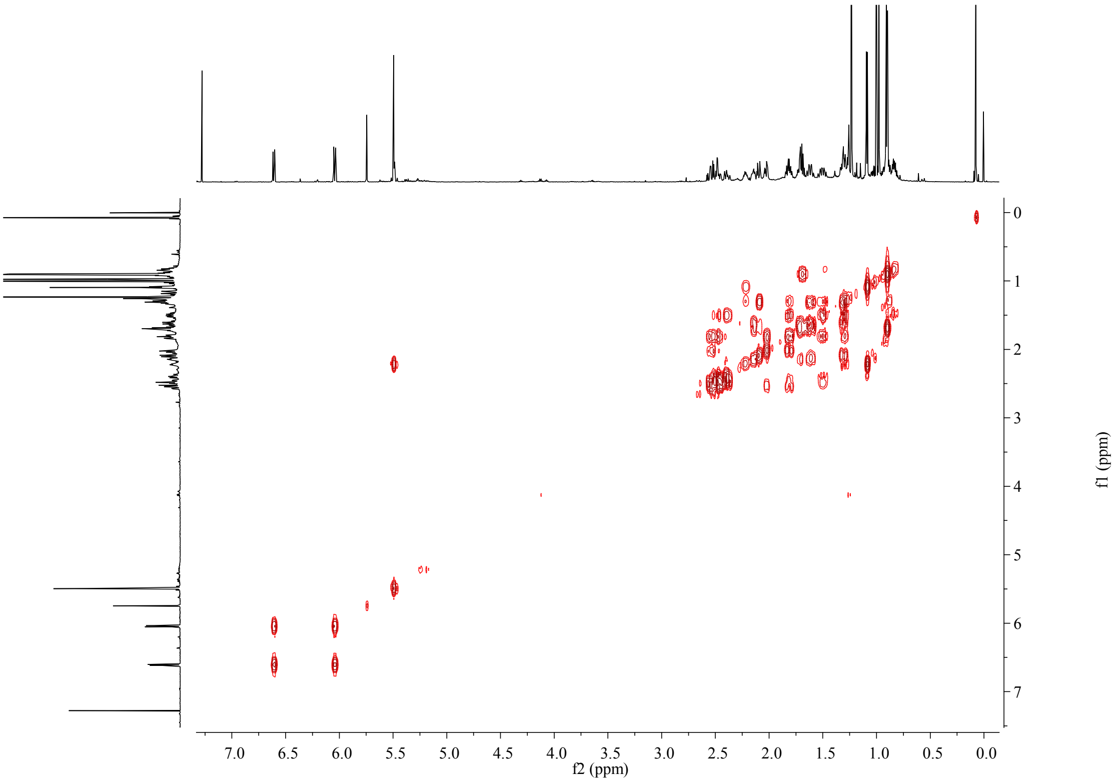

Figure S20. ^1H-^1HCOSY spectrum (600 MHz) of compound **6** in $CDCl_3$.

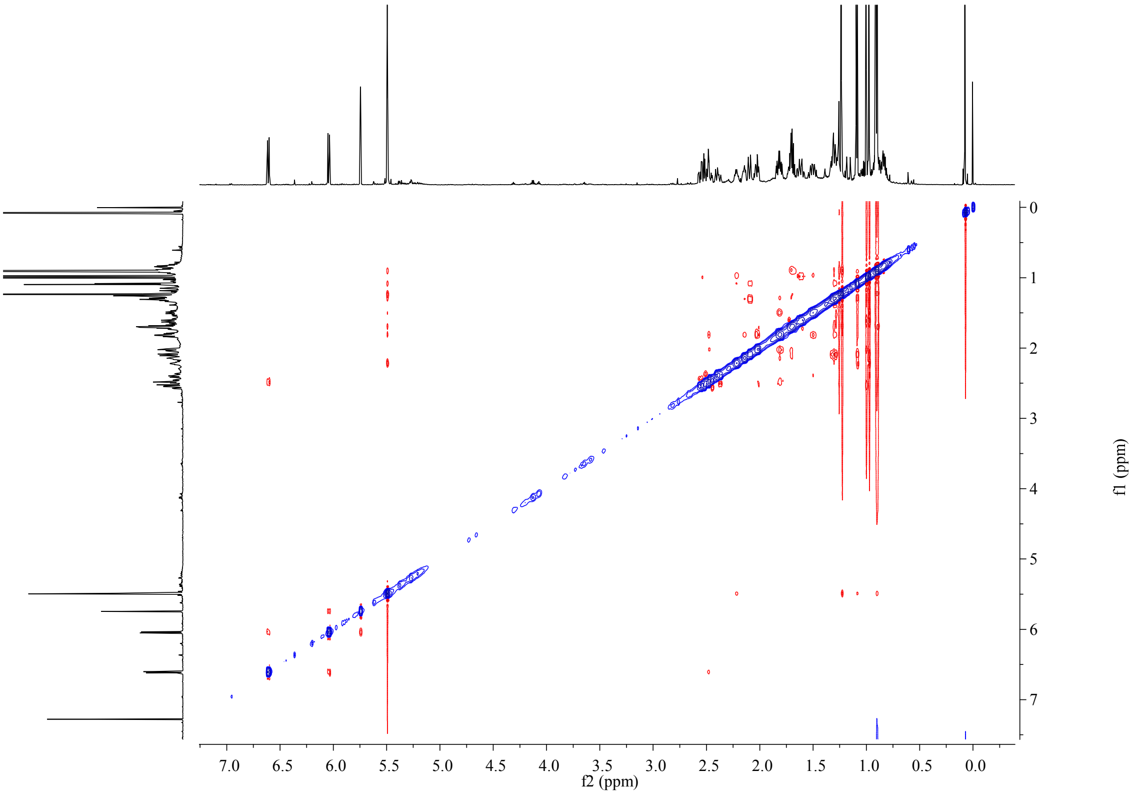

Figure S21. NOESY spectrum (600 MHz) of compound **6** in $CDCl_3$.

Mass Spectrum SmartFormula Report

Analysis Info

Analysis Name	D:\Data\MS\data\201603\liwensheng_kcn-7_43_01_1533.d	Acquisition Date	3/28/2016 4:22:21 PM
Method	LC_Direct Infusion_pos_100-1000mz.m	Operator	SCSIO
Sample Name	liwensheng_kcn-7	Instrument / Ser#	maXis 29
Comment			

Acquisition Parameter

Source Type	ESI	Ion Polarity	Positive	Set Nebulizer	0.4 Bar
Focus	Active	Set Capillary	4500 V	Set Dry Heater	180 °C
Scan Begin	100 m/z	Set End Plate Offset	-500 V	Set Dry Gas	4.0 l/min
Scan End	2000 m/z	Set Collision Cell RF	800.0 Vpp	Set Divert Valve	Waste

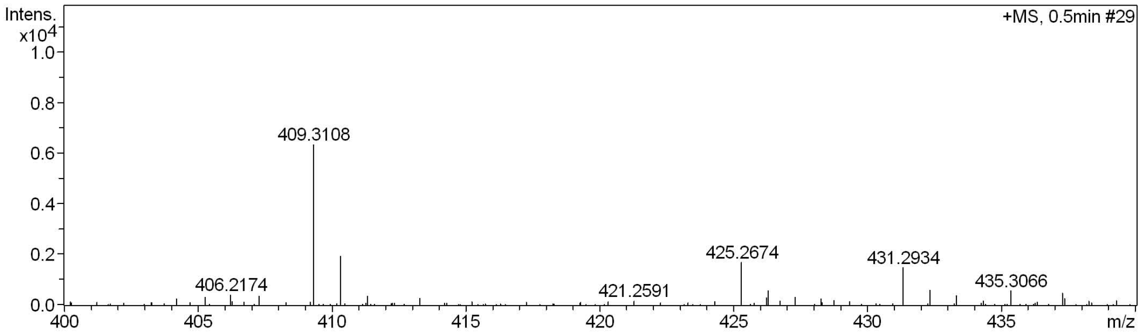

Meas. m/z	#	Formula	Score	m/z	err [ppm]	err [mDa]	mSigma	rdb	e¯ Conf	N-Rule
409.3108	1	C 28 H 41 O 2	100.00	409.3101	-1.7	-0.7	4.5	8.5	even	ok
431.2934	1	C 28 H 40 Na O 2	100.00	431.2921	-3.1	-1.3	110.0	8.5	even	ok

Figure S22. HRESIMS spectrum of compound **6**.
